# Decline and diversity in Swedish seas: Environmental narratives in marine history, science and policy

**DOI:** 10.1007/s13280-019-01247-1

**Published:** 2019-09-13

**Authors:** Susanna Lidström, Sverker Sörlin, Henrik Svedäng

**Affiliations:** 1 Division of History of Science, Technology and Environment, KTH Royal Institute of Technology, 100 44 Stockholm, Sweden; 2 Swedish Institute for the Marine Environment (SIME), Gothenburg University, Box 260, 405 30 Göteborg, Sweden; 3 Baltic Sea Centre, Stockholm University, 106 91 Stockholm, Sweden

**Keywords:** Baltic Sea, Declensionist narrative, Environmental framing, Environmental narratives, Marine environmental history, Swedish seas

## Abstract

Before the mid-twentieth century, there was no comprehensive narrative about empirical conditions in Swedish seas. Around 1970, this view changed profoundly. In line with growing research and the emergence of ‘the environment’ as a defining concept, conditions in Swedish seas were framed as a ‘narrative of decline’. Marine scientists have since recorded more diverse developments than are described by an overall declensionist narrative. Data show trends of interrupted decline, variability and even recovery, taking place at least partly in response to effective policy and legislation. We suggest that beyond the specialised fields of marine sciences and marine environmental history, the overarching narrative of decline has persisted, paying little attention to local and regional particularities as well as cultural and political dimensions of the marine environment. This overly uniform narrative risks obscuring historical reality and, hence, fails to adequately inform policy and the public about developments and outcomes of interventions in Swedish seas.

## Introduction: A narrative of decline

Knowledge of the status of fish stocks, other sea resources, vegetation and environmental conditions in the Swedish coastal seas was once fragmented and fragile. While local knowledge among fishermen and coastal communities have always been important, the perceived need to increase knowledge on a national scale is more recent. An early effort was made in the mid-eighteenth century, when the Royal Swedish Academy of Sciences showed interest in and promoted marine science, for instance by initiating a study on herring fishing in the Stockholm Archipelago (Humble 1745). The reason was an overwhelming negative deficit in trade of fish products, which concerned the Swedish government at the time and spurred several actions to improve the standards of the fishing industry (Awebro 2008). By the late 1800s, collection of data started to stabilise and become more systematic, and has improved continuously since then. Before the mid-twentieth century, there was no comprehensive narrative about the conditions recorded; variability was the overarching understanding and some long-term climatic trends on the centennial and millennial scales were observed (Hagen and Feistel 2005).

Between ca. 1950 and 1970, this view changed profoundly in line with growing marine and oceanographic research and more generally with the rise of a new understanding of ‘the environment’ as a certain entity that is measurable and with directional and definable ‘rates of change’. Based on this new understanding of the environment as a common name for a growing ‘catalogue’ of ‘problems’, including e.g. overpopulation, erosion, pollution, resource depletion and overfishing (Warde et al. 2018), the growing body of data on marine conditions was reframed as a ‘narrative of decline’. This was a global phenomenon, but it was also applied specifically to Swedish coastal seas. The narrative was particularly pronounced for the Baltic Sea but also comprised the North Sea and Skagerrak. The re-interpretation from variability to decline can be regarded as fully established by the early 1970s with main components including pollutants, especially hazardous substances, eutrophication and sea traffic issues, including oil spills (e.g. Dybern 1970; Jansson 1980).

With regard to marine biota, it has been known since ancient times that fishing and catch size affected fish stocks locally (Muscolino 2012). Real concern for the risk of undermining marine resources or even losing species is a much later phenomenon, though it was perceived as early as the eighteenth century, which saw the disappearance of the sea cow. Rudyard Kipling famously wrote about it in the story of Kotick, the white seal in *The Jungle Book* (1894), which was a critique of American stewardship (or lack of it) in Alaska, bought from Russia in 1867. Depletion of fish stocks became a matter of more widespread political and scientific concern with the arrival of trawl fishing in the North Sea in the late nineteenth century. ‘Overfishing’ as a scientific concept was introduced around that time, albeit first vaguely defined and much debated (Petersen 1900). In 1902, as a result of the concern for overfishing, the International Council for the Exploration of the Seas (ICES) was formed. In 1911, international cooperation to avoid extinction of the fur seal resulted in the North Pacific Fur Seal Convention, signed by the US, Russia, Japan and Great Britain. Minimal fishing in European seas during World War I demonstrated a recovery of fish stocks that reinforced the importance of previous human impact and as a result, demands on constraint and planning of harvesting were raised. Concepts such as total acceptable catch or ‘optimum catch’ were proposed to indicate that, just as in forestry where *Nachhaltigkeit* (sustainable yield) had been a norm since the eighteenth century, restraint and precaution were economically beneficial in fisheries as well (Sahrhage and Lundbeck 1974/1992, p. 288).

Ocean fishing went on unabated, however, and global annual harvests grew from less than 20 million tonnes after WWI to a peak of 90 million tonnes in 1988 (Muscolino 2012, p. 286). *The sea around us*, to quote the title of Rachel Carson’s 1953 widely read book, was still conceived of as too vast and too deep to be understood as anything but a dark, unknown and inhospitable wilderness where humans had barely started exploration, let alone wrought any significant havoc. In only a couple of decades, the master narrative of the ocean shifted completely, and evidence such as ever more frequent collapses of fish populations (sardines in Japan and California, cod in the Northwest Atlantic, herring in the Atlantic and the North Sea, anchovies in Peru, etc.) was mounting to suggest that the oceans were if not in immediate danger at least an integrated part of ‘the environment’, which was now understood as threatened and vulnerable.

The oceans were part of the concerns brought to the table at the United Nations Conference on the Human–Environment (UNCHE) in Stockholm in 1972, a safe sign of the new and concerned outlook (e.g. Patin 1982). November the same year, another UN conference in London adopted principles to regulate dumping of waste and pollution in the oceans (Schenker 1973). By then whaling and overfishing of, for instance, the Norwegian herring stock or the Peruvian anchovy had already added strength to a sense of decline in the seas. More recently, climate change, plastic pollution and fishing in general have been added to the narrative as drivers of global marine environmental degradation. This narrative of decline has been more or less hegemonic since the 1970s and has served as the essential understanding of legislation and regulation of the state of the seas. The understanding of conditions in Swedish coastal seas is to a considerable extent a reflection of this emerging and eventually pervasive global narrative, with the 1974 Helsinki Convention for the Protection of the Marine Environment of the Baltic Sea Area an important landmark. However, the Swedish narrative also has its particular features and certainly its own empirical foundations, with the Baltic Sea as an especially grave case in point, with an all but ‘dead’ sea floor (Elmgren 2001).

In recent years, marine scientists have recorded more diverse developments in Swedish seas than are described by an overall declensionist narrative. Data show trends of interrupted decline, variability and even recovery, taking place at least partly in response to effective policy and legislation, such as bans on several toxins (Nyberg et al. 2015). Nevertheless, beyond the scientific realm, an overarching declensionist narrative tends to persist. Elmgren et al. (2015) note that “The Baltic Sea is often portrayed as an environmental disaster area, by the media, by non-governmental environmental organisations, and by some scientists” (p. 339). Such a uniform declensionist narrative for Swedish seas is part of a much broader idea of global environmental degradation, which while important in some respects has also been criticised for downplaying historically and geographically specific environmental developments (e.g. Nixon 2011; Malm and Hornborg 2014), including outcomes of legislation and other forms of interventions—or lack thereof.

While many of the overarching traits of a declensionist framework remain indisputable, empirical data and historical analysis suggest that Swedish seas are better represented by a narrative of complexity with reform, relative stability and even recovery as strong elements. While acknowledging serious environmental impacts, past and present, a framework that recognises complex and divergent developments can showcase that many governmental interventions are and have been successful when and where they have been practised. Marine historian Bolster (2006) has emphasised the importance of situated and specific environmental narratives for the oceans, stating that “Central to such stories will be the recognition, common to some historians, that complex variables create historically specific situations – not universal ones, or replicable ones, or natural ones, but historically specific situations” (p. 582). In this paper, we suggest that such narratives are largely missing from public perceptions and policy frameworks for Swedish seas. Diverse trajectories seem overshadowed by a simplified narrative of overall degradation, pointing to a lack of societal reflection on specific developments in marine conditions. This may in turn counteract possibilities and prerequisites for an open dialogue on Swedish marine environments regarding what is attainable, at what costs and on what time scales. We illustrate this viewpoint with a few examples from influential policy frameworks, but our aim is also to highlight a need for further interdisciplinary research into how scientific findings and measurements of environmental conditions in Swedish seas are recorded and registered in broader society and non-scientific discourses—such research could provide a more substantial review and examination of Swedish marine environmental narratives than we are able to pursue within the scope of this article.

## Mixed messages: The empirical evidence

Some developments in Swedish coastal seas sustain a narrative of rapid and problematic change, even decline. Perhaps the area of most concern is climate change. Increased water temperature has been a major ecological driver in Swedish seas since the late 1800s. With inspiration from the famous British *Challenger* expedition between 1872 and 1876, the first hydrographic expedition in Swedish waters was conducted by R/V *Gustaf af Klint* in 1877. Soon these measurements became regular (Fonselius and Valderrama 2003). The time series show very clearly that bottom water temperatures have increased significantly over the twentieth century. Warmer climate conditions in the Baltic Sea have become especially pronounced since the 1980s, with a shift from a continental to maritime type of climate associated with large-scale changes in atmospheric circulation (Lehmann et al. 2011). Effects of higher temperatures are appreciable from records on ice cover in the Baltic Sea, Skagerrak and Kattegat, showing an apparent tendency of a reduction in ice cover over the last decades. While predictions of future climate changes and their consequences are uncertain, it is fair to say that climate and temperature records show a clear negative trend, and that the overall effects are cause for concern, in Swedish seas just like elsewhere.

Recent losses of eelgrass (*Zostera marina*) manifest another negative trend. However, the most disastrous effects occurred already in the 1930s, when almost all eelgrass in the North Atlantic died off due to a wasting disease (Rasmussen 1977). Before this episode, about half of the shallow Kattegat was covered in eelgrass meadows. Less than a fifth of this distribution area has been recovered, inevitably also due to decreased water transparency, restricting the distribution vertically.

In the Baltic Sea, eutrophication is a major ecological driver. Due to increased cultivation in the entire drainage area basin since the Middle Ages, the Baltic Sea, once oligotrophic with a rather low standing crop of fish and low productivity, has gradually become more enriched and hence more productive (e.g. Zillén and Conley 2010). During the nineteenth century, increased demand for agricultural products led to ever more intense cultivation (Hoffmann et al. 2000). Increased resource utilisation typically led to higher discharges of nutrients from farmed lands. However, elaborated techniques in handling manure and fertilisers have constrained discharges of nutrients in spite of a tremendous increase in productivity. Thus, quite counter-intuitively, nutrient discharges from farmed land in Sweden are still at about the same level as they were 150 years ago. Despite such counter-measurements, increased discharges from the Baltic Sea drainage area as a whole have caused increased eutrophication of the Baltic Sea since WWII; since about the 1990s, the trend has reversed in the southern Baltic Sea and Kattegat, as nutrient loading has eventually culminated and is presently decreasing (e.g. Andersen et al. 2017).

The combined effects of eutrophication and increasing bottom water temperatures have led to successively more severe hypoxia in the deeper parts of the Baltic proper due to higher respiration rates. Since 1900, the area of hypoxia in the Baltic Sea has increased from 5000 km^2^ to over 60 000 km^2^ (Carstensen et al. 2014). However, the distribution of hypoxic and anoxic bottoms has been rather stable over the last 40–50 years. A major inflow in the mid-1990s oxygenated bottoms in the Gotland deep temporarily but the deeper parts became hypoxic rather soon again. For now, hypoxia trends in Baltic coastal environments are mixed; improvements in oxygen content are clearly visible in the hypoxia-prone Stockholm Archipelago, whereas the situation is steadily getting worse in the Gulf of Finland. Hypoxia also leads to leakage of phosphorous from the sediments, and thus nitrogen becomes limiting for algae, promoting cyanobacteria growth (Zillén and Conley 2010).

Eutrophication is a complicated process and not entirely dependent on the availability of nutrients. Resilient ecosystems, including many trophic layers, are less likely to be disrupted by nutrient loading than simplified ecosystems. Trophic relationships within the Baltic, as well as in the Kattegat and Skagerrak Inshore, are indeed altered due to loss of predator fish biomass. Apex predators such as marine mammals and birds were initially also very common, but in the 1960s, high levels of toxic substances, mainly persistent organic pollutants, were found in Swedish waters and almost exterminated seal stocks in the 1970s. Other top predators such as sea eagles and peregrine falcons all but disappeared for similar reasons. As the contaminants have declined and hunting of seals became very restricted, marked recoveries have taken place (e.g. Olsson et al. 1994). The trend exemplified by the seal stocks is representative for overall conditions for hazardous substances in Swedish seas. A comprehensive study of toxins (PCBs, DDTs, HCHs and HCB) in marine biota in Swedish seas found that between 1969 and 2012 the levels decreased significantly, showing that measures taken in the form of bans and restrictions on different substances in the 1970s and 1980s have had the desired effect (Nyberg et al. 2015). While levels in the Baltic Sea remain elevated, with considerable variation between different substances and areas, the overall trend is one of recovery.

Another central dimension of Swedish seas are fisheries. Swedish fishermen who fished in offshore waters with longlines for large sized cod, ling (*Molva molva*) and other species in the Skagerrak and Kattegat were confronted at an early stage with falling catches. Lowering of the biomass level, especially the disappearance of big fish, forced artisanal Swedish fishers to explore new fishing grounds as their low-productive fishery became economically unsustainable already in the 1860s, unless the landings per unit effort was exceptionally high (Cardinale et al. 2014). As the longliners left the Kattegat and Skagerrak for outlying areas, demersal trawling and other active forms of fishing were introduced on the Swedish west coast at the beginning of the twentieth century. With the exceptions of the two world wars and a conspicuous rebound in the fish stocks during the so-called gadoid outburst in the 1970s, demersal stocks have tended to decline since then (Engelhard et al. 2016). The former, highly productive Swedish Skagerrak coast, as well as the Kattegat on the whole, has become some of the most degraded areas in the world in terms of predatory fish.

This continuous deterioration of the marine fish fauna along the Swedish coasts has, paradoxically, protected the fishing industry from being scrutinised (cf. Hultkrantz et al. 1997). Events during the 1980s, especially algal blooms, fish kills in connection to hypoxia in southern Kattegat and seal epizootic, which in the end turned out to be a natural phenomenon (e.g. Härkönen et al. 2006), contributed to a widespread beneficial view of fishermen as being part of a drama of ecological deterioration and dilapidation that they could not avert; they were victims rather than agents. This victimisation of a whole industry that after all had miniscule economic importance had consequences for sound fisheries management. As the enforcement of economic zones took place in the early 1980s, Swedish fishers traditionally fishing in the North Sea lost most of their former fishing opportunities. The simultaneous cod boom in the Baltic Sea partly solved the problem of excessive fishing capacity, as parts of the fishing fleet relocated there. The trawling limit, originally enforced in the early twentieth century to protect the coastal zone from over-exploitation, was also moved closer to the coast to present excess fishing capacity with new fishing opportunities (cf. Cardinale et al. 2014).

In the North Sea, biological conditions for fish stocks, especially gadoids, are similarly far from optimal, in spite of a strong recovery (ICES 2015). Recruitment has been depressed since around 2000, so the recovery is entirely due to reductions in fishing pressure. The biomasses of many fish species are nonetheless at record levels. In agreement with the Johannesburg Declaration on Sustainable Development, fishing has been downsized as to get higher yields, i.e. adjustments towards the biometric goal of maximum sustainable yield (MSY). Although MSY has been criticised for a long time (Larkin 1977; Finley 2011), this recovery still indicates that keeping to a restricted harvest strategy may turn stocks productive once again in spite of unfavourable conditions.

On the EU level, erratic decision-making marked fisheries governance during the first period of the common fishery policy (CFP) since its introduction in the mid-1980s, i.e. neglect of ICES catch advice, inadequate enforcement of rules, excessive subsidies to the fishing fleet and so forth (Sissenwine and Symes 2007). However, fishery management has improved considerably over the last decade. The most important measure has been to implement effort regulations since the turn of the century, whether in number of operating fishing vessels or number of days at sea (Fernandes and Cook 2013). Another important change is the introduction of the notion of ecosystem-based management, representing a more holistic approach to governance. However, while the idea has helped to shift perspectives, Elmgren et al. (2015) suggest that ecosystem-based management, while explicitly stated as a strategy for the Baltic Sea already in 1992, remains an idea more than a practice.

In light of these empirical examples, we suggest that there is a discord between the historical data of ecological trends in Swedish seas and a unified narrative of environmental decline: the eutrophication process in the Baltic Sea has been halted and is showing signs of an early phase of reversal, the contamination of several hazardous substances including oil spills are decreasing, and apex species such as eagle, cormorant, falcon and seal populations are recovering (e.g. Herrmann et al. 2014). This is not to diminish that, on the other hand, fisheries are not recovering according to expectations, sediments will continue to leak unhealthy compounds for many years, and plastic particles are accumulating. Above all, a warmer climate will put ecosystems under increasing stress, accelerating problems such as the oxygen deficit in the Baltic Sea.

## Diversity versus decline in policy frameworks

The empirical image of heterogeneous data lacking a uni-linear direction is in our view poorly represented in frameworks for Swedish marine policy. In an overview of successes and failures in the Baltic Sea, Elmgren et al. (2015) note that a narrative of declining or poor conditions tend to dominate in public and policy communications about conditions in Swedish seas; for example, a map produced by HELCOM in 2010 indicates bad environmental status in almost the entire Baltic Sea, failing “crucially, to present the real progress that has been made, proving that investing in the environment pays off” (p. 339). HELCOM’s earlier Baltic Sea Action Plan (2007, pp. 6–11) likewise fails to recognise past or partial improvements, such as decreased relative input of nutrients, focusing instead entirely on requirements needed to achieve the future ideal goal of “a Baltic Sea unaffected by eutrophication” by 2021; the vision of a final state of sustainability is emphasised at the expense of recognising situated and historical developments of continuously evolving human–environment relations. In HELCOM’s most recent “holistic assessment”, an introductory set of comprehensive parameters gives a similarly discouraging impression of overall environmental status as “not good”; a sub-basin specific overview suggests that in ten out of 17 basins there are no positive environmental trends at all, while in the remaining seven areas they are only minor improvements. The illustrations for both eutrophication and hazardous substances are also predominantly negative, in contrast to the detailed accompanying description which states that while hazardous substances continue to be a cause for concern “the number of improving trends outweighs the number of deteriorating trends” (HELCOM 2018).

A series of reports from the Swedish Agency for Marine and Water Management likewise communicates current conditions as overwhelmingly unfavourable. Part one in the series (2012) summarises that overall conditions in Swedish seas are not consistent with good environmental status, without recognising any positive developments. The detailed description of the status of seal populations neglects to mention successful reduction of toxins, stating only briefly that an increase in numbers has taken place since the 1970s when seals were threatened both by hunting and pollutants but that overall status remains poor. Rather than highlighting reduction of several toxins as an example of successful policy making and environmental improvement, recognition of positive trends for hazardous substances is not made until halfway through the report. The foreword to part four in the same series states that despite long-term and comprehensive attempts to improve the marine environment signs still abound that conditions are not good and negative pressures are increasing (2015), concluding that the goal of good environmental status in Swedish seas by 2020 will not be attained. Nowhere in the foreword or in the following summary are any improvements or cases of recovery mentioned. Overall, these reports give an overly negative impression of developments in Swedish seas and fail to communicate the positive impacts management and legislation demonstrably can have when such efforts are enabled and followed through.

These brief examples illustrate our impression that while academic disciplines such as marine sciences and marine historical ecology have made attempts to showcase and understand cases of recovery and improvement in Swedish seas, such efforts are much less noted and appreciated outside scientific contexts. Lack of coordination between itemised scientific trends and guiding frameworks for responsive policy are likely to have a negative impact on societal support for science-based marine policy. Overrepresentation of declensionist narratives may lead to poor public understanding and hence counteract informed debate regarding under which circumstances policies and other measures taken to improve environmental conditions may be successful, and what the reasons are for continued support and investment in such interventions.

Fisheries management is a case in point for how non-alignment between scientific recordings and public and policy frameworks can lead to misdirected management strategies. Translation of the complex developments of fish stocks and their causes into what may be considered thoughtful policy is often poor. Most decisions for Swedish fish stocks are made through the EU’s common fisheries policy, where negotiations showcase diverse priorities between different concerned member states. Recently decided 2019 fishing quotas for the Baltic Sea indicate limited responses to scientifically recorded trends; while numbers for cod in the eastern Baltic are strongly negative, quotas were only reduced 16% (however the fishery became closed in the second half of the year 2019), while tentative indicators of positive developments for western Baltic cod lead to a sudden jump in quotas with an increase of over 70%, despite estimates that overall current cod fisheries in the Baltic Sea are only 10% of what they were 30 years ago (ICES 2017). The press release from the European Council for the recent quotas indicates that the decisions are influenced by the persistent framing of EU fisheries as a predominantly socio-economic concern as much as an ecological and environmental one, stating that the new quotas represent a step towards achieving sustainability in Baltic Sea fisheries “whilst at the same time respecting the socioeconomic viability of our coastal communities”. This understanding is different from contemporary scientific frameworks for fisheries in for example the US, where the ecological sustainability of fisheries has been established as the primary concern and a basic legal requirement before any fishing can occur. The importance of the socioeconomic perspective persists in the EU notwithstanding the fact that fisheries in the Baltic Sea are of an insignificant value in an economic sense. Moreover, capital-intensive, highly industrial fisheries are systematically favoured by strong economic subsidies and higher quotas than the Baltic coastal fisheries which may soon have disappeared in large parts of the Baltic. The ‘costs’ of downsizing the fishing effort of industrial fisheries thus represent nothing less than gains, also for the local fishing villages (cf. Svedäng and Hornborg 2015).

## Appropriate narratives

Narratives are essentially sense-making devices—they frame scientific information and shape policy and public imaginations (e.g. Lakoff 2010). Often rooted in very old religious and philosophical tropes or patterns of societal temporalization, narratives of environmental decline have developed in recent decades into a dominant framework for understanding the relationship between people, societies and the natural world (e.g. Garrard 2012). The predominance of an overall narrative of decline is thus not unique to Swedish seas; in approval of coherence, broad-brush environmental narratives tend to favour simplicity over complexity and regularly fail to reflect the details of sometimes diverging directions of social and ecological change, as well as of scientific data and methods. Mixed messages, regional varieties, and far-reaching and drawn out environmental processes that are difficult to make sense of—to narrate—are often underrepresented in policy as well as public imaginations (Nixon 2011).

Moreover, similarly to climate change, ocean environments are particularly challenging to perceive because they are invisible and inaccessible to most people—we see the shore and patches of the surface, the rest is hidden. In order to know and care about what happens in the sea we depend on information and narratives provided, directly or indirectly, by scientists (e.g. Chiarappa and McKenzie 2013). Recognition of the problem of “shifting baselines” (Jackson et al. 2011) originated in marine sciences and is especially relevant for the oceans, where many things are “invisible to the human eye yet absolutely essential for understanding the marine past” (Taylor 2013, p. 68). Recent efforts have been made to remedy the lack of knowledge about historical marine conditions, including in the Baltic Sea, for example, through the History of Marine Animal Populations (HMAP) Project (e.g. Lajus et al. 2013; MacDiarmid et al. 2016).

The preference for simplicity and generalisation is apparent in some of the most compelling environmental narrative concepts, such as the “death of nature/end of nature” (e.g. McKibben 1989), or more recently those of “planetary boundaries” (Rockström et al. 2009) or the “Anthropocene” (Crutzen and Stoermer 2000). The most overarching and influential of all these narrative concepts has probably been that of “the environment” itself, which has remained a defining idea for about 70 years (Warde et al. 2018). There are also numerous examples of collapse stories from the modern history of the oceans that apply broad scale tropes and concepts of environmental decline and harmful human impact on marine ecosystems (e.g. Speer 1997).

These concepts are obviously meaningful. They serve as convenient shorthand for certain overarching tendencies and phenomena within an infinitely complex earth system and an almost endless set of social–ecological relationships and their changes over time. They also function as providers of instrumental functions—heuristic, explanatory, warning and mobilising. But they also have shortcomings which need to be recognised and discussed. Criticised for downplaying local and regional particularities as well as cultural, political and other human dimensions (e.g. Malm and Hornborg 2014), such concepts risk enforcing an overly uniform narrative of environmental degradation, including regarding the state of Swedish seas. In fact, we argue that the overarching decline story has obscured historical reality of Swedish marine conditions and also that it has, hence, not adequately informed policy and the public. Overarching perspectives may overshadow successful and constructive efforts to manage and improve conditions on local and regional scales. The creation of marine protected areas is a case in point. The ban on trawling in Öresund, for example, has shown how local initiatives on limited geographical scales can have very positive effects, despite perceptions of marine life as highly mobile and interconnected (e.g. Højgård Petersen et al. 2018). Persuasive narratives can also be counterproductive by leading to premature conclusions. For example, concerns about toxins and eutrophication in Swedish seas formed a strong public narrative in the 1980s and 1990s. This narrative also became the dominant framework for understanding declines and changes in fish stocks, and as a result, the role of fishing practices was downplayed. Not until the 2000s was overfishing rather than toxins appreciated as the main reason for the decline of ground-fish on the Swedish west coast in particular, and relevant regulations called for (Svedäng 2003). A challenge is therefore to carefully differentiate between times when overarching uni-directional narratives are appropriate, and other times when local or otherwise specific conditions show results that are not in line with dominant trends, and thus better understood through other frameworks.
